# Benthic Ecosystem Calcification Measured with Coupled pH and O_2_ Aquatic Eddy Covariance

**DOI:** 10.1021/acsestwater.5c00481

**Published:** 2026-04-21

**Authors:** Dirk Koopmans, Allison Schaap, Volker Meyer, Paul Färber, Lauren Queiss, Luis Montilla, Socratis Loucaides, Soeren Ahmerkamp, Ulisse Cardini

**Affiliations:** † 42254 National Oceanography Centre, Southampton SO14 3ZH, U.K.; ‡ Max Planck Institute for Marine Microbiology, Bremen 28359, Germany; § Department of Integrative Marine Biology (EMI), Stazione Zoologica Anton Dohrn, Naples 80122, Italy; ∥ 28389 Leibniz Institute for Baltic Sea Research Warnemünde, Rostock 18119, Germany; ⊥ Department of Integrative Marine Biology (EMI), Stazione Zoologica Anton Dohrn−National Institute of Marine Biology, Ecology and Biotechnology, Genoa Marine Centre, Genoa 16126, Italy

**Keywords:** seagrass, epiphytes, photosynthesis, community, CO_2_ vent, alkalinity

## Abstract

We present a method to quantify benthic ecosystem calcification from simultaneous pH (proton) and O_2_ eddy covariance flux measurements. In benthic ecosystems, photosynthesis is a proton sink, while calcification is a proton source. Where calcification is the dominant nonmetabolic proton source, it can be isolated as the residual between the measured proton flux and the flux predicted from O_2_-derived metabolism. We demonstrate this technique in *Posidonia oceanica* seagrass meadows near Ischia, Italy, where coralline algae epiphytes are the primary calcifiers. The method resolved a diurnal calcification signal consistent in magnitude with previous estimates for seagrass epiphytes. However, our pH measurements and proton fluxes also revealed widespread, diffusive CO_2_ vent influence at both the vent-adjacent site and the control site (670 m away), demonstrating that control sites near natural CO_2_ vents may not provide the stable baseline often assumed. Excluding the vent-affected data removed substantial portions of the data set, resulting in high uncertainty, while also illustrating the insights that high-speed multiparameter sensing provides. Our error analysis identifies accuracy in pH, alkalinity, and the ecosystem photosynthetic quotient as critical constraints on this and other pH-O_2_ based calcification measurements, particularly in environments where calcification rates are small relative to metabolic fluxes.

## Graphical abstract

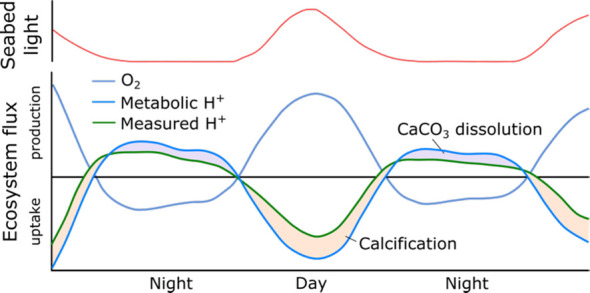

## Introduction

Ocean acidification and marine heat waves pose threats to many benthic calcifying marine organisms. Ocean acidification has caused scleractinian coral calcification to decline 7–30%.^1–3^ Future acidification will exacerbate this stress, and may be especially detrimental to coralline algae.^4^ Coralline algae abundance in *P. oceanica* seagrass meadows at a naturally occurring CO_2_ vent declines to near zero with a pH decrease of just over 0.2 pH units.^5^ Across CO_2_ vents and species, their abundance declines by 50% (a mean across species and sites) with a pH decrease of just 0.13 units.^6^ For scleractinian corals, however, a more immediate threat is marine heat waves. Climate change-driven marine heat waves are occurring with greater frequency and duration,^7^ and are causing major losses of corals worldwide.^8,9^

To improve our understanding of the response of corals and coralline algae to these stressors, in situ measurements of their growth are needed. The most common technique for measuring coral and coralline algae growth, as calcification, under in situ hydrodynamic conditions is the alkalinity anomaly technique.^10,11^ Alkalinity is the sum of proton acceptors over donors^12^ and in natural seawater it is primarily composed of bicarbonate (HCO_3_^–^) and carbonate (CO_3_^2–^) ions. Calcification in seawater occurs as a combination of the following two reactions^13^

$${\mathrm{Ca}}^{2+}+2\mathrm{HC}O_{3}^{-}⇌\mathrm{Ca}{\mathrm{CO}}_{3}+{\mathrm{CO}}_{2}+H_{2}O$$ 1a

and

$${\mathrm{Ca}}^{2+}+\mathrm{HC}O_{3}^{-}⇌\mathrm{Ca}{\mathrm{CO}}_{3}+H^{+}$$ 1b

In both equations two moles of alkalinity are consumed for every mole of CaCO_3_ formed (in eq 1b one mole of HCO_3_^–^ is consumed and one mole of protons is produced, lowering alkalinity by two moles). The alkalinity anomaly technique quantifies calcification from this change in alkalinity, so it is only accurate in environments where calcification is the most important alkalinity-affecting process. For in situ measurements, net calcification (*G*_net_) is calculated as

$$G_{\mathrm{net}}=\frac{\Delta A_{t}\rho z}{2\tau }$$ 2

where Δ*A*_*t*_ is the change in total alkalinity, ρ is seawater density, *z* is the mean depth of water, and τ is the residence time of water in contact with the reef.^14^ A limitation of the alkalinity anomaly technique is τ, which must be approximated. As a result of uncertainties in the technique, a cross-study comparison of in situ measurements of calcification with it revealed no relationship between *G*_net_ and the percent cover of calcifiers in coral reefs.^15^ The limitations of the technique obscure insights into calcification.

Boundary layer flux techniques, i.e., eddy covariance and gradient flux, solve the residence time uncertainty of the alkalinity anomaly technique, and, through pH and O_2_ measurements, can be used to quantify calcification. Aquatic eddy covariance^16^ quantifies flux as

$$\mathrm{flux}={\overset{-}{w}}^{\prime }{\overset{-}{c}}^{\prime }$$ 3

where *w*′ is the fluctuating, turbulent, component of vertical water velocity, *c*′ is the fluctuating component of solute concentration, and the overbar indicates an arithmetic mean over time. pH and O_2_ eddy covariance fluxes have previously been measured together,^17,18^ but have not been applied to calcification. The gradient flux technique,^19,20^ on the other hand, has been shown to be able to measure ecosystem calcification.^21^ It quantifies turbulent flux as a diffusivity term acting on the concentration gradient, i.e.,

$$\mathrm{flux}=-K_{z}\frac{\partial C}{\partial z}$$ 4

where *K*_*z*_ is the vertical turbulent diffusivity and $\frac{\partial C}{\partial z}$ is the vertical gradient in solute concentration in the benthic boundary layer (i.e., in the bottom meter of the water column). *K*_*z*_ is estimated as a function of hydrodynamic quantities.^22^ Alkalinity sensors do not yet have the precision to resolve the concentration differences typically required for $\frac{\partial C}{\partial z}$ , but Takeshita et al.^21^ demonstrated that the gradient flux technique could be used to determine *G*_*net*_ from measurements of pH and O_2_, following carbonate system calculations by Barnes.^23^ Calcification is a source of protons. This is true whether calcification follows eq 1a or eq 1b, generating protons or CO_2_, respectively, because CO_2_ equilibration in water generates protons as follows

$${\mathrm{CO}}_{2}(\mathrm{aq})+H_{2}O⇌H_{2}{\mathrm{CO}}_{3}⇌H^{+}+\mathrm{HC}O_{3}^{-}⇌2H^{+}+{\mathrm{CO}}_{3}^{2-}$$ 5

Takeshita et al.^21^ revealed the light dependency of net ecosystem calcification (NEC) for two coral reef sites, and found that environmental changes in pH (from 7.9 to 8.1) had little effect on calcification rates. However, an uncertainty in gradient flux measurements is the parametrization of *K*_*z*_. In environments with high bed roughness it can lead to large biases.^24^ One cause of this is flow deformation around local roughness elements which intensify vertical transport.^25^

In the present study, our primary goal was to test if coupled pH and O_2_ eddy covariance could be used to determine ecosystem calcification. For this, we deployed a pH and O_2_ eddy covariance system in a Mediterranean seagrass (*Posidonia oceanica*) meadow in late summer, when the abundance of epiphytic calcifiers was at their peak. A secondary goal was to examine the sensitivity of these epiphytes, primarily coralline algae, to acidification. For this, we conducted the study at a CO_2_ vent and at a nearby control site. We chose a CO_2_ vent meadow where wind-driven reversals in along-shore currents would carry the CO_2_ vent plume either through the CO_2_ vent meadow or away from it, alternately exposing the meadow to low-pH waters that were enriched in dissolved inorganic carbon (DIC), or to background pH. Ecosystem proton fluxes and calcification were determined at background pH.

## Materials and Methods

### Study Site

We conducted this study at the island of Ischia, Italy in September of 2020 at the Chiane del Lume vent (40° 42.918′ N, 13° 58.013′ E; Figure 1). We did not determine vent sulfide concentrations but submarine vents at Ischia all have similar chemistry,^26^ and those studied previously do not contain sulfide.^5,27^ At Chiane del Lume, hundreds of streams of fine CO_2_ bubbles rise through bare sands over an area of approximately 20 m by 10 m.

**Figure 1:**
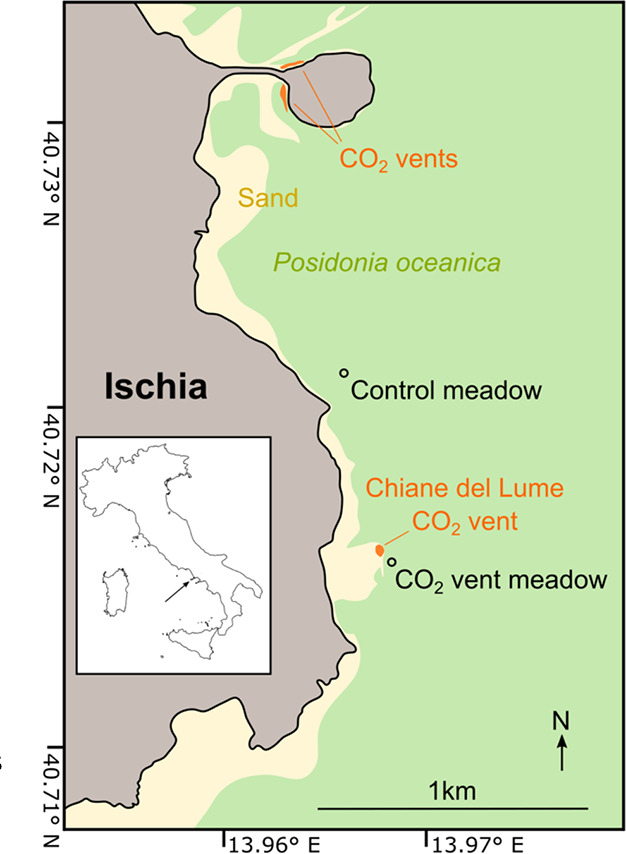
Map of the study area near the Chiane Del Lume vent at the island of Ischia, Italy. Seagrass coverage was estimated from satellite imagery and is less complete than indicated. Inset map by NordNordWest/Wikipedia (CC BY-SA 3.0 DE license).

In a preliminary dive, we found that calcifying epiphytes were abundant in meadows around the CO_2_ vent, even where the seagrass grew as close as 5 m from it. This was contrary to our expectation that epiphyte abundance would be noticeably lowered close to a CO_2_ vent. We followed the prevailing along-shore currents and designated a meadow 40 m SSE of the vent as “the CO_2_ vent meadow.” We designated one 670 m to the north as the control meadow. The depths were 13 and 8.5 m, respectively. We chose a shallower depth for the control meadow to elevate it above the shoaling of the thermocline by internal waves, which did not affect our results (indicative temperature data included in Figures S1 – S3). There was no visual difference in calcifying epiphyte cover between the CO_2_ vent meadow and the control meadow. Epiphytes were abundant at both. We measured eddy covariance fluxes at the CO_2_ vent meadow from 18 to 23 September 2020, and at the control meadow from 23 to 25 September 2020.

### Environmental Sensing

Turbulent water velocities were determined at 16 Hz with an acoustic Doppler velocimeter (ADV; Nortek AS). The details of the custom eddy covariance pH sensing technology have been presented previously.^18^ Briefly, following earlier work,^17^ we determined pH using an Ion Sensitive Field Effect Transistor (ISFET; Microsens SA), which we placed in an opaque housing to exclude light. Water was pumped from the measurement volume of the ADV through a mini flow cell that contained the ISFET and thermistor, then past an Ag/AgCl reference electrode fitted with a ceramic membrane to reduce stirring sensitivity.^18^ The ISFET signal was amplified 10-fold, linearized, and temperature-compensated. The amplified ISFET signal was 590 mV per pH unit, and based on this relationship, the ISFET pH was single-point calibrated to in situ pH measured by lab-on-chip (see below). Finally, a custom gear pump reversed flow direction past the ISFET for 1 min out of every 30 min to eject debris from the pumped flow path. For eddy covariance O_2_ sensing, we used high-speed minisensor optodes (UHS-O2-Sub with FSO2 Subport, Pyroscience, GmbH) operated at a frequency of 5 Hz. The oxygen sensors were calibrated in O_2_-saturated and anoxic water in the laboratory prior to deployment. Oxygen concentrations were calculated from Δ*φ* (shift of the emitted vs excited wavelengths of light) following a Stern–Volmer relationship.^28^

For eddy covariance measurements and other supporting measurements, we mounted instruments to lightweight fiberglass frames (Figure 2). We positioned the ADV measurement volume 115 cm above the bed, this was about 65 cm above the canopy. We aligned the tips of the O_2_ minisensor and the pH intake tube 27 and 32 mm from the measurement volume to minimize their acoustic backscatter.^29^ The pH intake tube drew water 40 cm from the measurement volume to the ISFET sensor. That was further than ideal, but it minimized the risk of corrosion of ISFET wiring if seawater creeped in. Dispersion within this tube slowed the 90% signal response (*t*_90_) of the ISFET from 1.2 to 2.4 s. We deployed two identical eddy covariance frames, each equipped for pH and O_2_ flux measurement. On one system, inconsistencies in pumped flow caused variations in the pH signal, obscuring ecosystem pH fluxes. We include only the pH fluxes from the system with the more consistent flow, but we present O_2_ fluxes measured with both systems.

**Figure 2:**
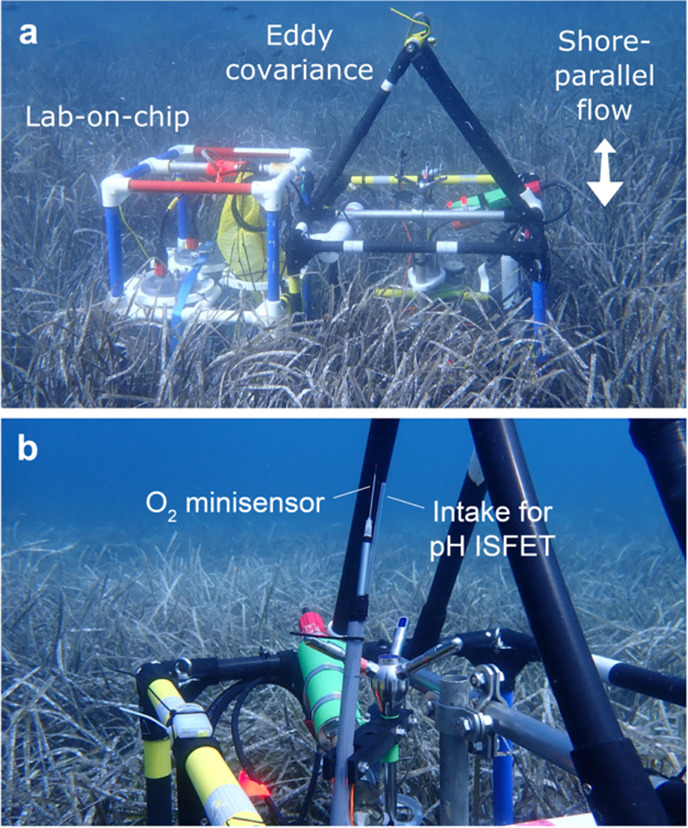
(a) Instruments deployed in the control meadow with the eddy covariance frame aligned for shore-parallel flow. (b) Close-up of the eddy covariance O_2_ minisensor and pH ISFET intake tubing at the measurement volume of the acoustic Doppler velocimeter.

We characterized the carbonate chemistry using in situ measurements of alkalinity and pH (total scale) with lab-on-chip sensors developed by the National Oceanography Centre. Both lab-on-chip sensors implement spectrophotometric assays for the determination of pH. The pH sensor uses meta-cresol purple dye,^30,31^ and the alkalinity sensor uses bromophenol blue dye.^32,33^ The alkalinity sensor determines pH after mixing the sample with a known volume of acid and degassing the resulting mixed sample. Sample alkalinity is calibrated against certified reference materials (CO_2_ Seawater Reference Materials, Scripps Institute of Oceanography, batches 162 and 180) also analyzed in situ. The accuracy of the alkalinity sensor is 10 μmol kg^–1^. The accuracy and precision of the pH lab-on-chip are 0.005 and 0.003 pH units, respectively. Two sample inlets were used on both lab-on-chip sensors so that we could measure each analyte at two heights (20 and 80 cm), within and above the seagrass canopy. pH lab-on-chip measurements were made continuously (every 19 min) during every deployment, while alkalinity measurements were only made continuously (every 12 min) during one deployment. The mean alkalinity during that deployment (2650 μmol kg^–1^) was assumed throughout the experimental deployments at Ischia.

We deployed additional sensors for supporting measurements. Multiparameter loggers (Virtuoso, RBR ltd.) were equipped with galvanic O_2_ sensors (OxyGuard, RBR ltd) to determine concentration differences within and above the seagrass canopy (at 20 and 80 cm above the bed). We corrected for a 10 μmol L^–1^ offset of one sensor by matching it to the concentration recorded by the other when water velocities were at their greatest. At those times, mixing between the canopy and overlying water minimized concentration differences. This adjustment allowed us to identify differences of less than 5 μmol kg^–1^. The multiparameter loggers above the canopy were also equipped with glass electrodes (pH AMT 6000 m, Analysenmesstechnik *GmbH*), which we calibrated to lab-on-chip pH. A photosynthetically active radiation (PAR) sensor (Odyssey, Dataflow Systems Ltd.) was positioned above the canopy on the instrument frame. A vertical profile of temperature sensors (HOBO Pendant, Onset Computers, Inc.; at 5, 20, 80, 95, 135 cm) was used to identify thermal stratification. Finally, seagrass shoot density per square meter, determined from 5 random placements of 24 cm × 24 cm quadrats, was 350 ± 110 and 360 ± 70 at the CO_2_ vent and control meadows, respectively.

### Eddy Covariance Data Processing

Eddy covariance data were downsampled to the frequency of optode sampling (5 Hz) for data processing in Matlab following previously published methods.^34^ Briefly, we applied a planar fit to the current velocity field to correct for the tilt of the ADV.^35^ We determined fluxes in 29 min intervals (excluding the 1 min pump flow reversals). We decomposed the 5 Hz time series into mean and fluctuating components using a running average of 500 s to minimize losses of low frequency flux contributions.^36^ We corrected for the time lag between velocity measurement and solute concentration measurement by aligning them at their highest correlation within a short time window (±2 s). Following flux calculations, we examined the linearity of cumulative flux contributions over time to identify fouling, sensor impairment, and issues due to water mass changes. Three percent of the data were affected and they were removed from time series calculations. To quantify high frequency contributions to proton and O_2_ fluxes, and to examine if turbulence was adequate at low water velocities, we examined fluxes in frequency space as cospectra.^37^ Data were exported to OriginPro for plotting and data interpretation.

### Calculation of Ecosystem Calcification

Following Barnes^23^ the effect of ecosystem processes on temporal changes in water column DIC can be expressed in terms of net photosynthesis (*P*_net_, a DIC sink) and net calcification (*G*_net_, also a DIC sink) as

$$\Delta {\mathrm{DIC}}_{\mathrm{water}\,\;\mathrm{column}}=-\Delta {\mathrm{DIC}}_{\mathrm{Pnet}}-\Delta {\mathrm{DIC}}_{\mathrm{Gnet}}$$ 6

where DIC_water column_ is the net effect of ecosystem processes on water column DIC. In terms of proton fluxes, the above equation can be represented as

$${flux\,\;H}_{e\cos\mathrm{ystem}}^{+}=-{flux\,\;H}_{\mathrm{Pnet}}^{+}+{flux\,\;H}_{\mathrm{Gnet}}^{+}$$ 7

where *flux* H_ecosystem_^+^ is the flux of protons from the ecosystem to the water column. *flux* H_Pnet_^+^ has a negative sign because photosynthesis removes protons, while *flux* H_Gnet_^+^ has a positive sign because calcification produces protons. We then calculated *flux* H_Pnet_^+^ as

$$flux\;H_{\mathrm{Pnet}}^{+}=\left(O_{2}\;flux\frac{1}{Q}\right)\frac{{\mathrm{dH}}^{+}}{\mathrm{dDIC}}$$ 8

where O_2_ *flux* is the eddy covariance oxygen flux, *Q* in the light is the net community photosynthetic quotient (PQ, which is defined as ΔO_2_/ΔCO_2_), and in the dark is the inverse of the net community respiratory quotient (RQ, which is defined as ΔCO_2_/ΔO_2_). dH^+^/dDIC quantifies proton production by the carbonate system, at equilibrium, per unit (e.g., 1 μmol kg^–1^) of DIC taken up. The term inside parentheses represents *P*_net_. We determined the ratio dH^+^/dDIC as a function of time-varying pH and temperature (alkalinity held constant) using numerical perturbation of CO2SYS^38^ (*k*_1_ and *k*_2_ dissociation constants calculated with the Lueker et al.,^39^ refit of Mehrbach;^40^ *k*_SO4_ of Dickson et al.;^41^ *k*_F_ of Perez and Fraga;^42^ and the borate to salinity ratio of Lee^43^). We used this ratio to express calculated proton fluxes in CO_2_ equivalents, i.e., *flux* CO_2 equiv._ = *flux* H_wat. col._^+^(dDIC/dH^+^). *Q* is largely a function of the C:N ratio (*n*) of the organic matter that is fixed and respired and can be estimated as $\frac{n+2}{n}$ .^44^ We approximated the C:N, and *Q*, of the ecosystem as follows. Epiphytes have a C:N of ∼13.7,^45^ while seagrass leaves have a C:N of ∼26.8.^46^ Epiphytes contribute an average of one-third of *P. oceanica* meadow photosynthesis and respiration at this time of year.^47^ Therefore, a proportionate ecosystem C:N ratio is 22.2, for a *Q* of 1.09. We assumed the same *Q* for light and dark measurements.

Similar to *P*_net_, *G*_net_ can also be expressed in terms of an H^+^ flux as

$$flux\;H_{\mathrm{Gnet}}^{+}=G_{\mathrm{net}}\frac{{\mathrm{dH}}^{+}}{d(\mathrm{DIC}\,\;2A_{t})}$$ 9

where *G*_net_ is net ecosystem calcification per unit area per time, and dH^+^/d­(DIC 2A_t_) quantifies proton production per unit of CaCO_3_ fixed. As with dH^+^/dDIC, we determined the ratio as a function of time-varying pH and temperature using numerical perturbation of CO2SYS (by subtracting 1 μmol kg^–1^ DIC and 2 μmol kg^–1^ alkalinity). pH changes during our study had a greater effect on this ratio than measured changes in alkalinity (Figure S4). We used the resulting pH change, at equilibrium, to find dH^+^/d­(DIC 2A_t_). In a further step, we quantified the sensitivity of calculations of calcification to inaccuracies in measured pH, alkalinity, and *Q*, both for our method and for the similar method of Takeshita et al.^21^ (Supporting Information, Appendix A).

Using eqs 7, 8, and 9, we can then solve for *G*_net_ as a function of H^+^ and O_2_ fluxes as,

$$G_{\mathrm{net}}=\left[H^{+}\;flux-\left(O_{2}\;flux\frac{1}{Q}\right)\frac{{\mathrm{dH}}^{+}}{\mathrm{dDIC}}\right]\frac{d(\mathrm{DIC}\,\;2A_{t})}{{\mathrm{dH}}^{+}}.$$ 10

We then calculated daily net ecosystem calcification (NEC) as

$$\mathrm{NEC}=\overset{®}{G_{\mathrm{net}\;\mathrm{light}}\;}\frac{h_{\mathrm{light}}}{24}+\overset{®}{G_{\mathrm{net}\,\;dark}\;}\frac{h_{\mathrm{dark}}}{24}$$ 11

where *G*_net light_ and *G*_net dark_ refer to measurements in the light and dark, *h*_light_ and *h*_dark_ to the number of light and dark hours per 24-h day. Finally, ecosystem respiration (R), gross primary production (GPP), and net ecosystem metabolism (NEM) were determined from oxygen fluxes using established techniques.^48^ For seagrass meadows, changes in O_2_ storage (i.e., changes in the concentration of dissolved O_2_ in the meadow over time) are important for quantifying ecosystem fluxes.^49^ However, we only made this correction to O_2_ fluxes at the specific times mentioned below. We did not account for changes in proton storage. Both pH and O_2_ concentration were affected by vent CO_2_ enrichment, making accurate corrections for changes in ecosystem-driven storage impossible during those times. Throughout this manuscript, except where noted otherwise, errors are reported as standard errors. These were propagated from light and dark measurements to net ecosystem measurements as quadratic means.

## Results and Discussion

### Vent CO_2_-Enriched Flow

Both the CO_2_ vent meadow and the control meadow were acidified by vent CO_2_-enriched waters. At the CO_2_ vent meadow, CO_2_ enrichment (i.e., pH reduction) occurred primarily in flow from the west (Figure 3), though it also occurred in flow from the southwest (Figure S1). During these events, pH commonly fell by 0.1 unit, consistent with dissolved inorganic carbon (DIC) enrichment of 65 μmol kg^–1^. However, dissolved oxygen only fell by 10–20 μmol kg^–1^ (Figures S1 and S2). The greater DIC enrichment than O_2_ drawdown suggests that the enrichment was due to vent CO_2_, not respiration. At the control meadow, vent CO_2_ enrichment occurred in flow from the northwest. The pH reduction was smaller, 0.02–0.06 units (Figure 4), and dissolved oxygen concentrations were unaffected by these events (Figure S3). Additionally, during the final 6 h of measurements at the control meadow, the pH dropped as low as 7.94 while current velocities were minimal (<2 cm s^–1^). As a result of this extended low-pH event, the mean pH at the control meadow (7.98) matched that of the CO_2_ vent meadow during the time series shown in Figures 3 and 4.

**Figure 3:**
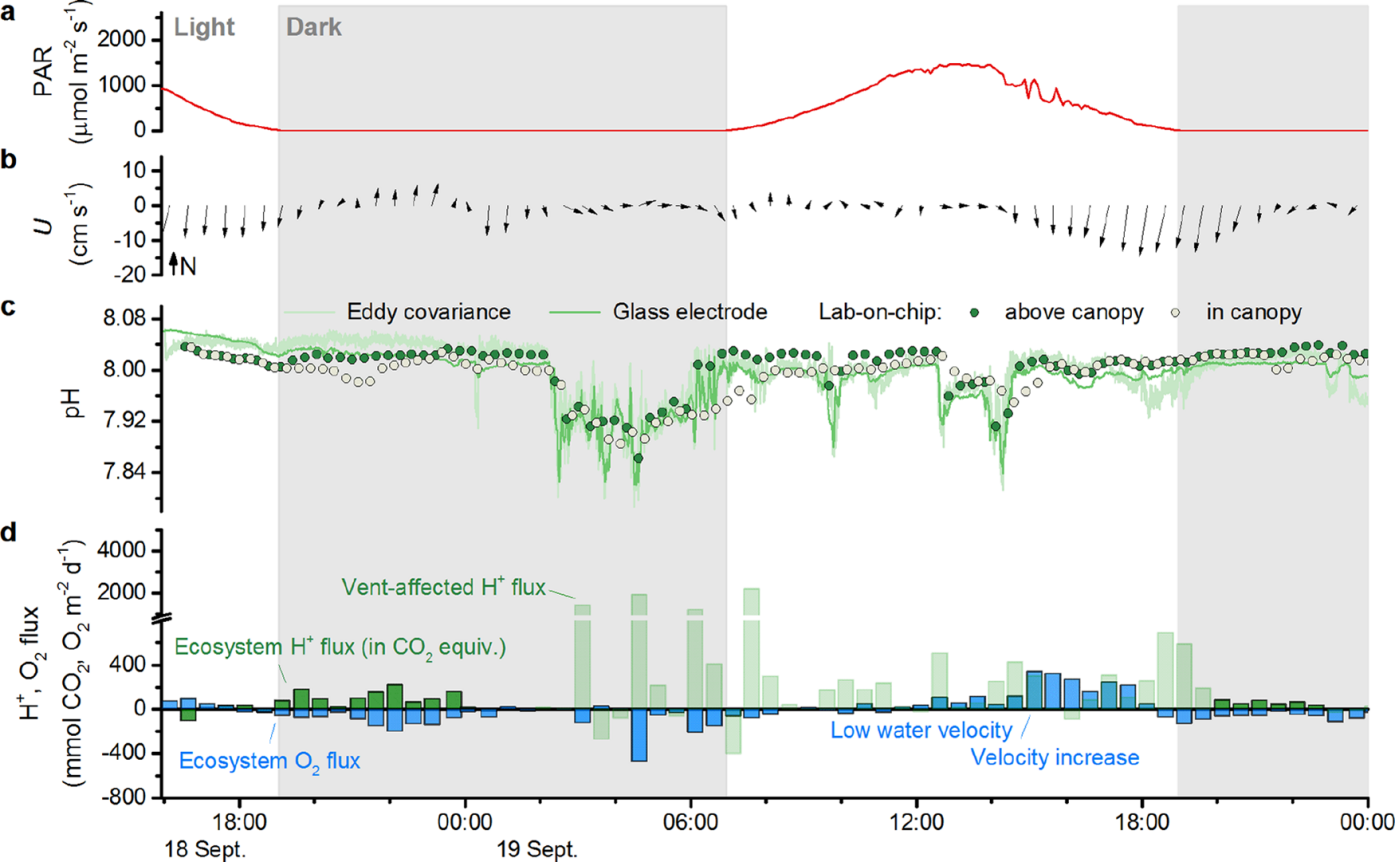
CO_2_ vent meadow time series of (a) PAR, (b) current vector with respect to north, (c) pH recorded by the eddy covariance ISFET, a glass electrode, and a lab-on-chip sensor, which through a second inlet also recorded pH within the canopy. (d) Flux of protons (in CO_2_ equivalents) and O_2_. Color-saturated bars represent ecosystem fluxes. Desaturated bars represent vent-affected fluxes.

**Figure 4:**
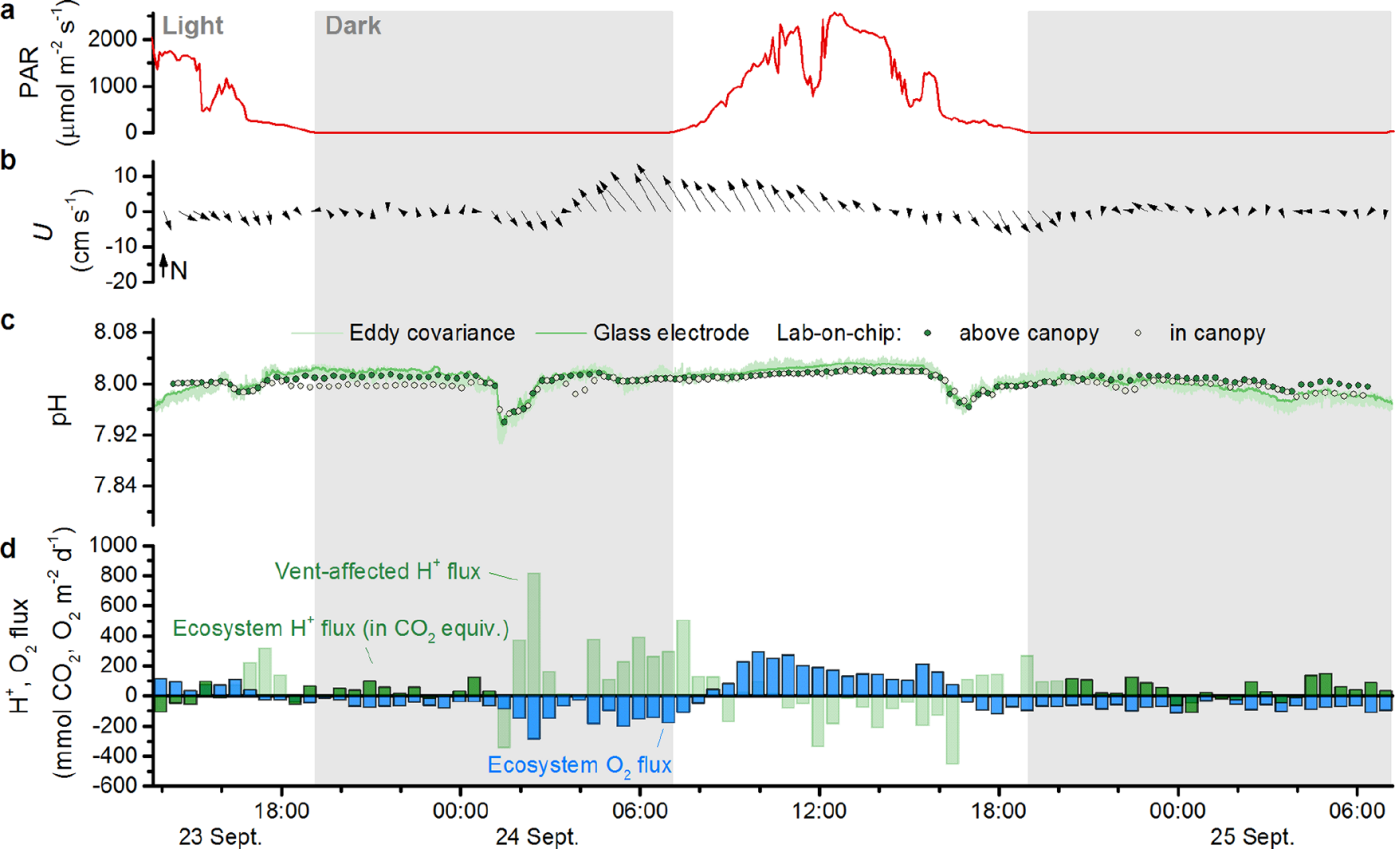
Control meadow time series measurements (a–d) as presented in Figure 3.

### Proton and O_2_ Fluxes

At Ischia, large effluxes of protons caused by vent CO_2_-enriched waters occurred at both the CO_2_ vent meadow and at the control meadow. At both meadows, these occurred in flow from multiple directions away from the known vent, and occurred in the presence and absence of CO_2_ enrichment. At the CO_2_ vent meadow, proton flux reached 2000 mmol CO_2_ equivalents m^–2^ d^–1^ during low-pH flow from the west (Figure 3b–d). Proton flux also reached 600 mmol CO_2_ equivalents m^–2^ d^–1^ while at background pH (8.04) in flow from the northeast. At the control meadow, proton flux reached 800 mmol CO_2_ equivalents m^–2^ d^–1^ during low-pH flow from the west and northwest (Figure 4b–d). Proton flux also reached 500 mmol CO_2_ equivalents m^–2^ d^–1^ while at background pH in flow from the southeast. We classified these large proton fluxes as “vent-affected” given their magnitude in comparison to the O_2_ fluxes presented below. The source of vent CO_2_ at both meadows was likely its upward diffusion through sandy sediments, as has previously been observed at Ischia meadows as far as 50 m from known CO_2_ vents.^50^

At the CO_2_ vent meadow and the control meadow, oxygen flux responded mainly to changes in light, though it was also affected by changes in water velocity and, to a lesser extent, by the presence of CO_2_-enriched waters. At the CO_2_ vent meadow, oxygen was produced during the day and taken up at night. Production was over 250 mmol m^–2^ d^–1^ during the afternoon of 19 September (Figure 3). However, at similar irradiance in the hours just prior to this, oxygen production was only one-fifth as great. The difference appeared to be caused by changes in water velocity which increased from about 2 cm s^–1^ to over 4 cm s^–1^. O_2_ depletion in CO_2_-enriched waters, present just prior to the increase in velocity, may have also contributed. At the control meadow the maximum daytime production was similar. At night, an increase in velocity also appeared to drive an increase in oxygen uptake. Oxygen uptake increased from 60 to 150 mmol m^–2^ d^–1^ as water velocities increased from close to 2 up to 10 cm s^–1^ during the night of 24 September (Figure 4).

Outside of the large proton fluxes at both meadows described above, proton fluxes were generally consistent with diel cycles of photosynthesis and respiration. At the CO_2_ vent meadow, on the first afternoon and into the night, net proton uptake and production were generally similar in magnitude but opposite in sign to O_2_ production and uptake (Figure 3d; 18 Sept.). A low-pH event then caused high proton fluxes, but during the final night, proton production was again similar in magnitude to O_2_ uptake. At the control meadow, on the first afternoon and into the night (excluding a brief, low-pH event), net proton uptake and production were also generally similar in magnitude but opposite in sign to O_2_ production and uptake (Figure 4d; 23 Sept.). However, through the early hours of 24 September and into the day, consistent currents from the southeast appeared to elevate proton fluxes. That morning, O_2_ production was substantial but there was little net uptake of protons, indicating a source of CO_2_ that was great enough to offset photosynthetic inorganic carbon uptake. At the end of the day, following a change in current direction, net proton production was again similar in magnitude but opposite in sign to O_2_ uptake.

Based on the above observations we discriminated “vent CO_2_-affected” proton fluxes from “ecosystem” fluxes, i.e., those due to metabolism, calcification, and CaCO_3_ dissolution. We use the term “vent” to refer to any geological source that enriched the meadows in CO_2_, even if not from the Chiane del Lume vent. Finally, we observed an additional pattern of nighttime CO_2_ enrichment of both meadows that is worth noting. As water velocities dropped below 2 cm s^–1^, the pH in the canopy typically dropped relative to the pH above it. Examples are just after sunset on 18 September at the CO_2_ vent meadow (Figure 3c,d) and at sunset on 23 September at the control meadow (Figure 4c,d). In both cases the decrease in pH was consistent with a DIC enrichment of the canopy on the order of 10 μmol kg^–1^. At these times, the dissolved oxygen concentration in the canopy was almost equal to the dissolved oxygen concentration above it (Figures S1 and S3). Nevertheless, because these differences in DIC were not inconsistent with nighttime respiration, we classified this enrichment as due to the ecosystem, as opposed to vent CO_2_. Perhaps the best evidence that this CO_2_ enrichment was metabolic, or at least had minimal effect on proton fluxes, is that a net uptake of residual protons (consistent with CaCO_3_ dissolution) was observed during this CO_2_ enrichment at the control meadow.

### Observations of Ecosystem Calcification and Metabolism

At the CO_2_ vent meadow, after excluding vent-affected proton fluxes, only 3 h of daylight observations remained with which to determine *G*_net_. During those hours, net calcification was 28.9 ± 23.4 mmol CaCO_3_ m^–2^ d^–1^ (*n* = 6 half-hour intervals; Figure 5b). At night, there was also net calcification, but at half the daytime rate (15.4 ± 12.9 mmol CaCO_3_ m^–2^ d^–1^; *n* = 18). At the control meadow, during 3.5 h of daylight, net calcification was 25.4 ± 33.8 mmol CaCO_3_ m^–2^ d^–1^ (*n* = 7; Figure 5d). The first night, there was net dissolution (−15.9 ± 13.7 mmol CaCO_3_ m^–2^ d^–1^; *n* = 12). During the second night, dissolution doubled in magnitude (−38.9 ± 14.5 mmol CaCO_3_ m^–2^ d^–1^; *n* = 22). The cause of this difference was unclear. pH fell during the second night at the meadow, but the peak rate in dissolution occurred while pH was similar to the first night.

**Figure 5:**
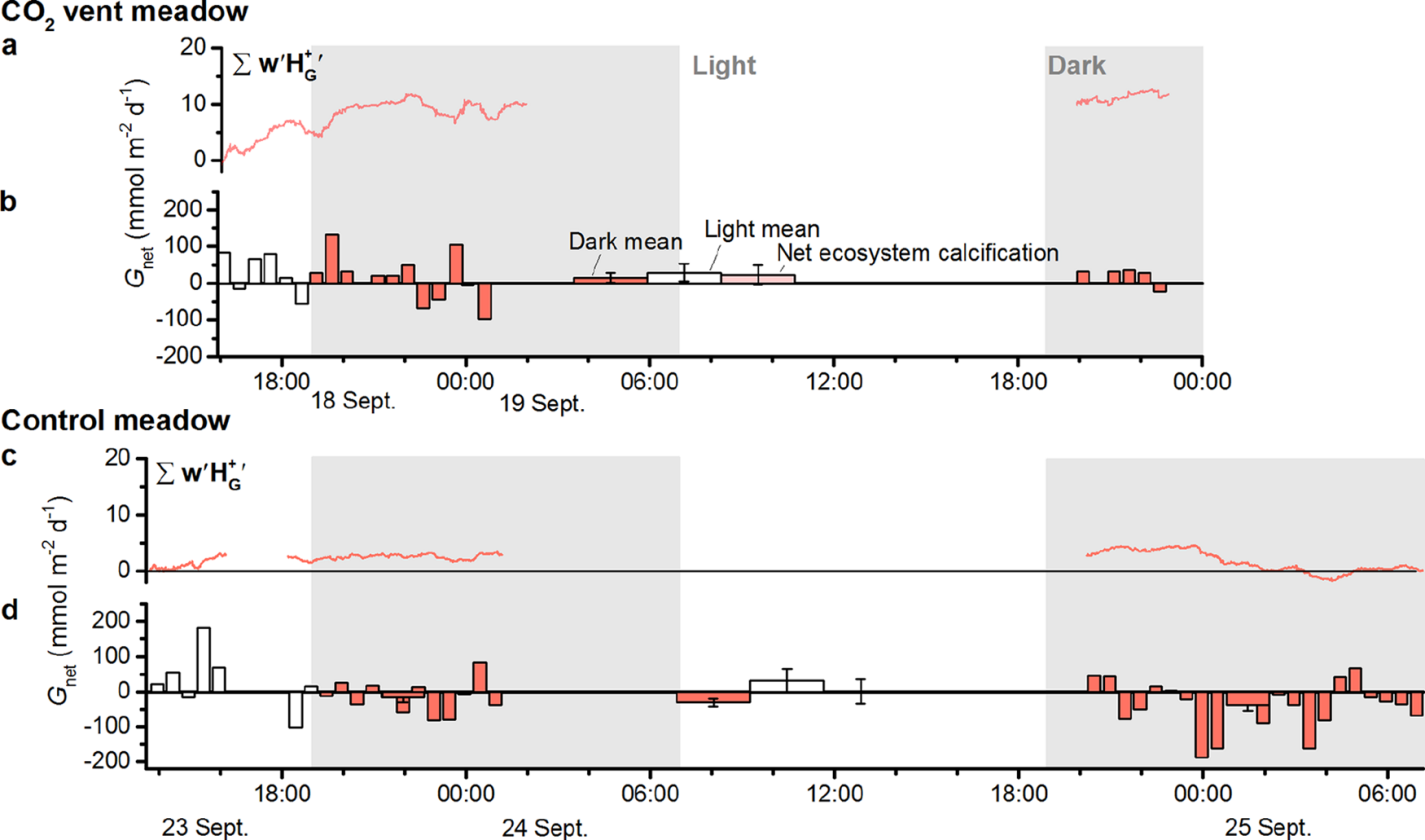
Residual proton flux due to calcification and CaCO_3_ dissolution at (a,b) the CO_2_ vent meadow, and at (c,d) the control meadow. Net ecosystem calcification (NEC) was (b) 22.1 ± 26.8 mmol CaCO_3_ m^–2^ d^–1^ (s.e., *n*_Light_ = 6 and *n*_Dark_ = 18) and (d) −2.7 ± 35.4 mmol CaCO_3_ m^–2^ d^–1^ (*n*_Light_ = 7 and *n*_Dark_ = 34).

Net ecosystem metabolism was generally heterotrophic or near a metabolic balance, but there was substantial variability. We quantified R, GPP, and NEM at the CO_2_ vent meadow over one partial day (18 September) and three full days. We quantified them at the control meadow over one full day. The CO_2_ vent meadow was net heterotrophic on three of the 4 days of observations (Figure 6). On the remaining net autotrophic day (20 Sept.), high photosynthetic production occurred while irradiance was similar to the preceding day but daytime water velocities were 75% greater. This result appeared to be robust. Dissolved oxygen storage did not contribute to it, and the observed oxygen fluxes were generally reproducible (Figures 6c and S5). Our assessment of this and of other results is presented further below.

**Figure 6:**
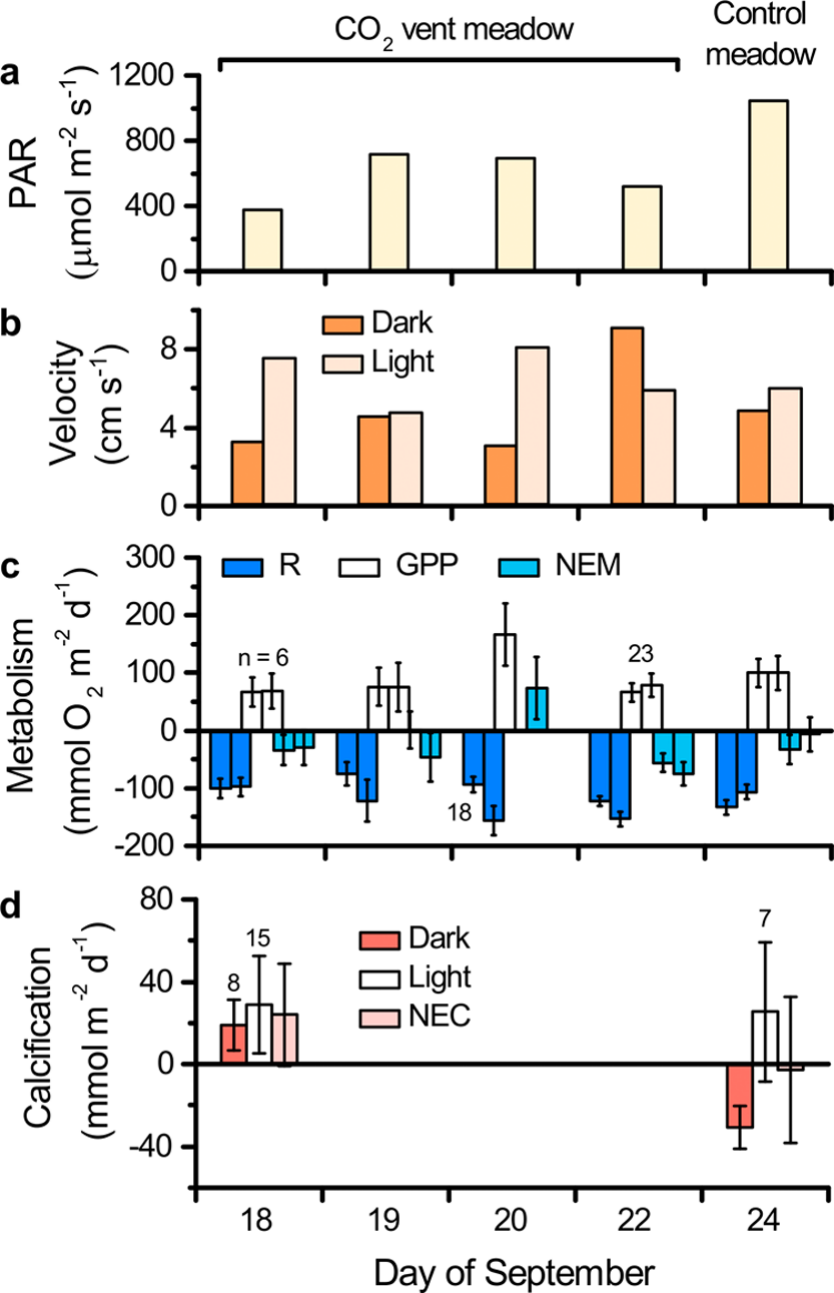
Drivers of organic carbon metabolism in the seagrass meadows over 4 days of measurements. (a) PAR, (b) mean water velocity, (c) respiration (R), gross primary production (GPP), and net ecosystem metabolism (NEM; determined with two O_2_ eddy covariance systems), and (d) net ecosystem calcification in the dark, in the light, and total (NEC; *n* = 24 half-hour observations in panels (c,d) except where noted).

### Assessment of Theory

We investigated the propagation of measurement errors through our calculations of calcification and found that they become increasingly important as the ratio of *G*_net_ to *P*_net_ becomes small. Errors in pH, alkalinity, and *Q* cause inaccuracies in calculated *G*_net_ to increase exponentially as the ratio of *G*_net_ to *P*_net_ decreases below 0.5 (Figure 7). For pH, at a *G*_net_ to *P*_net_ ratio of 0.2, a 0.04 unit error in pH would cause a 40% error in *G*_net_ (Figure 7a) due to errors in the assumed ratio of dH^+^/dDIC (eq 10). For *Q*, at a ratio of 0.5, a 10% error in *Q* would cause a 30% error in *G*_net_ (Figure 7b) due to errors in the assumed effect of *P*_net_ on proton flux. These inaccuracies also apply to other methods that use changes in dissolved O_2_ and pH to determine net calcification, such as Takeshita et al.^21^ (Figure 7b).

**Figure 7:**
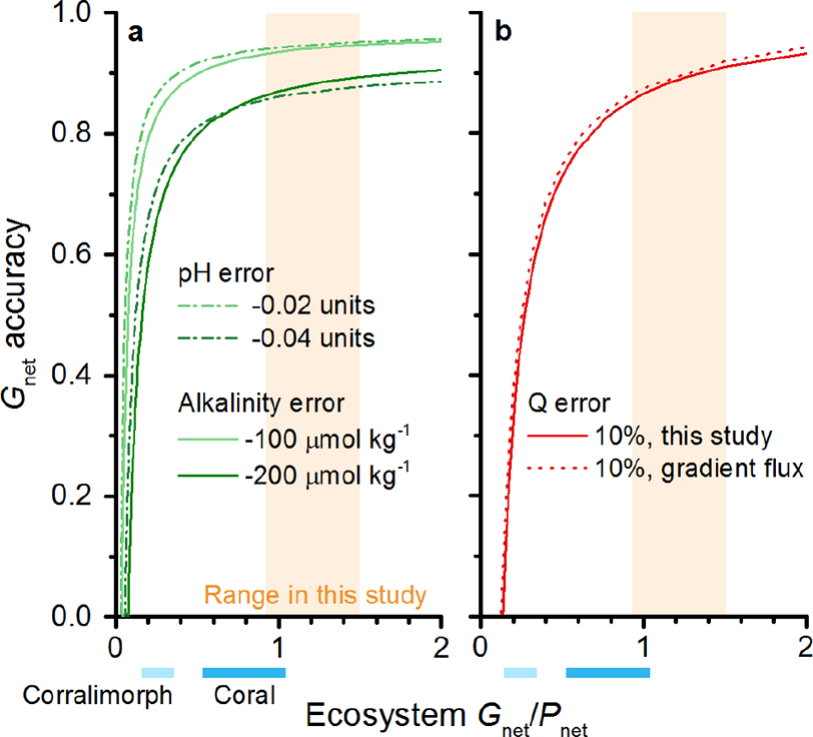
Analysis of the propagation of measurement errors. Underestimates in calculated *G*_net_ as a function of ecosystem *G*_net_/*P*_net_, with sensitivity to errors in (a) pH and alkalinity and (b) in the photosynthetic quotient (this study and pH and O_2_ gradient flux^21^). In beige: daytime *G*_net_/*P*_net_ for this study (mean ± s.e., *n* = 13). At bottom: daytime *G*_net_/*P*_net_ at corallimorph and coral reef sites.^21^

The range of *G*_net_ to *P*_net_ at which these techniques are sensitive to error coincides with the range of *G*_net_ to *P*_net_ in many ecosystems of interest. In a healthy coral reef site, daytime observations of the ratio of *G*_net_ to *P*_net_ commonly fall in the range of 0.5–1.0 ^21^ (range indicated at bottom of Figure 8). This is similar to the daytime ratio of *G*_net_ to *P*_net_ at a coral recolonization site^21^ (also at bottom of Figure 8). At night, the ratio diminishes as net CaCO_3_ dissolution occurs. As a result, the diurnally integrated ratio of NEC:NEM in coral reefs can commonly be as low as 0.2.^51^ In carbonate reef sands, the ratio of daytime calcification to photosynthesis can be 0.15–0.5; and of nighttime respiration to CaCO_3_ dissolution 0.1–1.0.^52^ In a *P. oceanica* meadow, the diurnally integrated ratio of NEC to NEM can vary widely, but similarly low values occur.^53^ In this study, the mean daytime *G*_net_ to *P*_net_ was 1.20, but lower ratios would be expected in seagrass meadows with less abundant epiphytes and in *P. oceanica* meadows at other times of year.

**Figure 8:**
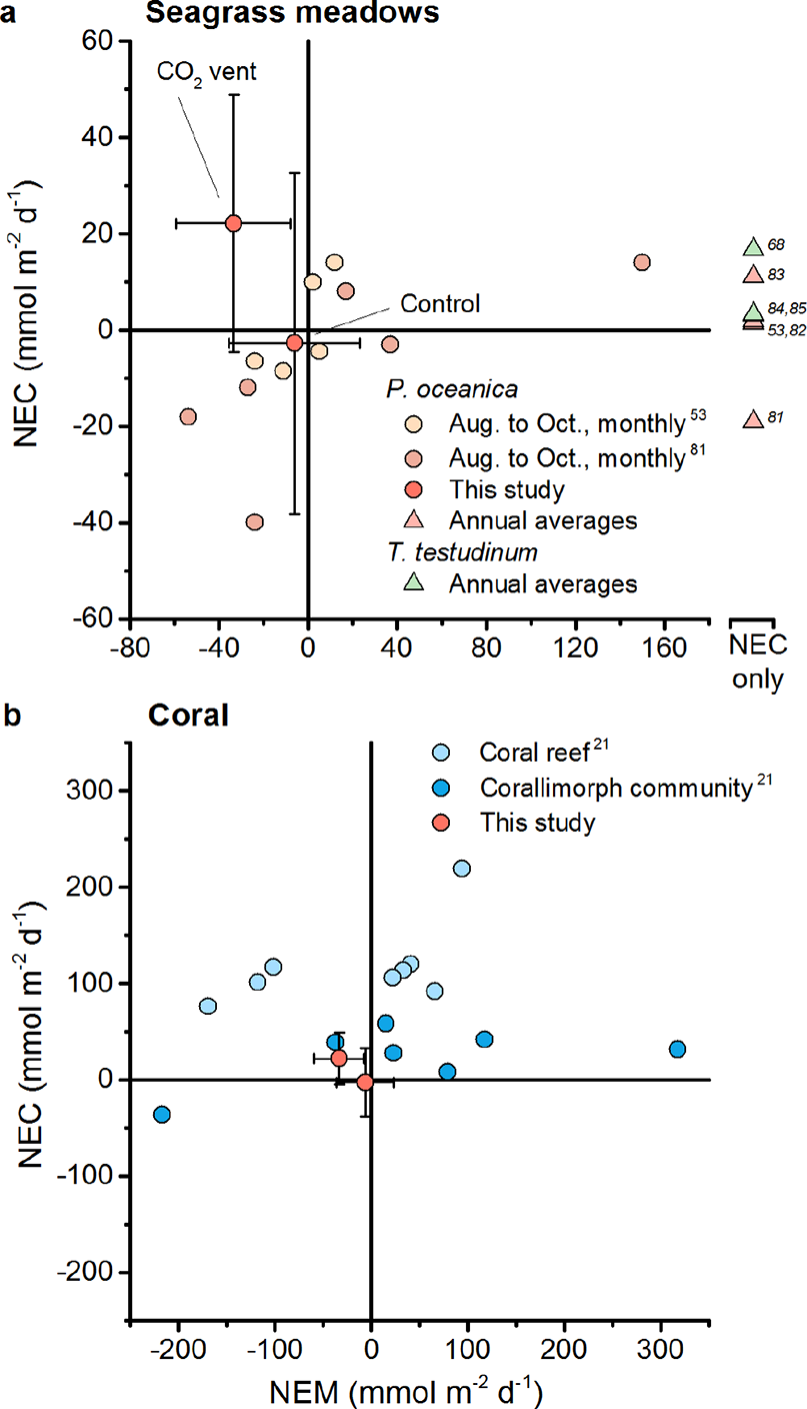
Comparison of NEC across studies and ecosystems. (a) Seagrass meadow NEC as a function of NEM determined monthly from August to October, and NEC as annual averages (at right). (b) Coral reef NEC as a function of NEM determined daily with the pH and O_2_ gradient flux technique.^21^ The uncertainties associated with cited data are not shown.

Based on the ratios of *G*_net_ to *P*_net_ in coral reef sites and *P. oceanica* meadows, and the sensitivity of calculations of *G*_net_ to the measurement errors of Figure 7, accurate calculation of *G*_net_ may require a pH accuracy of ±0.02 units, an alkalinity accuracy of ±100 μmol kg^–1^ and 90% or better accuracy in the assessment of *Q*. Ultimately, the required accuracies will be a function of *G*_net_ to *P*_net_ in the ecosystem. In future studies, a better understanding of *Q*, and of *G*_net_ to *P*_net_, could be gained by using benthic chambers to quantify relative changes of O_2_, pH, and alkalinity in a subset of the ecosystem.

### Assessment of Proton Fluxes

The pH eddy covariance sensor needs to be fast and precise enough to resolve the range of frequencies of turbulence that contribute to fluxes. To assess whether this criterion was met, we can compare the turbulent cospectra resolved by the “ideal” sensor (O_2_) against the slower one (pH).^37^ We did observe a small loss of higher frequency contributions in our proton fluxes (Figure S6). Interestingly, this appeared to be more of an issue during the day, when ∼20% of the flux contributions were lost than at night, when less was lost. The day-to-night differences may be due to vertical differences in photosynthesis and respiration in the canopy. Photosynthesis occurs primarily at the top of the canopy,^54^ while respiration (including that of shaded leaves, rhizomes, and sediment) occurs primarily within the canopy. Therefore, higher frequency and smaller-scale, above-canopy turbulence generated by drag against the top of the canopy may carry a greater portion of the ecosystem flux signal during the day than they do at night. The greater loss of daytime proton fluxes than nighttime ones means that our measurements underestimated daytime calcification by a small amount. If we increased daytime proton fluxes by 20% to account for this error, the net effect on calcification would be less than 15%, inconsequential for this study. This smaller-than-expected effect is because net dissolution also occurred during some daytime intervals.

The loss of high frequency signals in our pH flux measurements was due to two factors. One, we used a 40 cm-long intake tube. Fluid dispersion in this tube slowed the effective sensor response time. Two, shared power with the O_2_ minisensor introduced noise to the pH signal. For future work, the sensor can be operated with a shorter intake tube and with its own battery power.

In these calculations, we assumed that carbonate system equilibrium has been reached for parcels of water at the eddy covariance measurement volume. This is a reasonable assumption. CO_2_ equilibrates rapidly in warm water. At the water temperature at Ischia (25 °C), 90% of carbonate system equilibration occurs 35 s after CO_2_ is introduced.^55,56^ We used prior model output^57^ to estimate that in Ischia meadows, the median solute transit time from a leaf at the top of the canopy, through its diffusive boundary layer, and in turbulent transport to the measurement volume, is 240–550 s. This is long enough to allow equilibrium to also be reached at much lower temperatures. For example, at 8 °C the 90% equilibration time is 118 s.

In these calculations, we also assumed that the second-most important driver of proton fluxes, after photosynthesis and respiration, was calcification and CaCO_3_ dissolution. Researchers have previously applied a tightly related assumption, that calcification is the primary driver of changes in alkalinity, to quantify calcification in seagrass meadows,^53,58,59^ and have applied this assumption extensively in coral reefs.^14^ A simplifying assumption that applies to alkalinity budgets at ocean margins is that the net contribution of alkalinity to seawater by anaerobic processes is limited to net denitrification and pyrite burial.^60^ However, because we measured proton (and O_2_) fluxes over time, time-varying rates of these and other processes may affect our measurements. The effects of biogeochemical processes on proton fluxes can be calculated as the product of the carbonate system-dependent buffer factor and the change in charge of acid–base species.^61^

The median calcification rate during light and dark conditions at Ischia was 30 mmol m^–2^ d^–1^ (Figure 6d). This corresponds to a change of −0.034 pH units day^–1^ for the roughly cubic meter of water below the measurement volume. Per mole of reaction, denitrification produces protons at one-fourth the rate of calcification.^61^ Therefore, it would need to occur at four times the rate that we attributed to calcification to have a similar effect. Instead, its rates in oligotrophic seagrass sediments are close to 1–3 mmol m^–2^ d^–1^.^62^ Unlike denitrification, dissimilatory nitrate reduction to ammonia (DNRA) is strongly alkalinizing. Following Soetaert et al.,^61^ its effect on proton uptake is 65% greater than CaCO_3_ dissolution. Rates of DNRA have not been measured in oligotrophic seagrass sediments, but can be constrained by gross rates of nitrate uptake, which are comparable to rates of denitrification.^62,63^ Therefore, the potential impact of DNRA on proton fluxes in this environment is limited. Similarly, the impact of nitrification is constrained, and limited by, very low gross rates of ammonium uptake (<1 mmol m^–2^ d^–1^).^62,64,65^

The primary anaerobic process in *P. oceanica* sediments is sulfate reduction, which occurs at rates up to 12 mmol m^–2^ d^–1^.^66^ This rate represents the net oxidation of organic matter by fermentation and subsequent oxidation of its products by sulfate reduction, which is tightly coupled to it.^67^ We can use the buffer factor and mean charge of acid–base species to quantify the effect of these processes on pH under given seawater–carbonate system conditions (Supporting Information, Appendix B). Taking the major anaerobic reactions involved, the first step is fermentation, followed by the oxidation of H_2_ and, e.g., acetate via sulfate reduction. These coupled reactions are a net source of protons of just over half that of oxic organic carbon respiration. The “missing” component of the proton flux is generated with the oxidation of sulfide. Combined, the net effect of the coupled fermentation-sulfate reduction reactions and the subsequent oxidation of reduced sulfides, has precisely the impact on proton (and O_2_) fluxes as that of oxic organic carbon respiration. This is consistent with the finding that in an oligotrophic seagrass meadow denitrification and sulfate reduction are only minor sources of alkalinity, and that CaCO_3_ production was its primary sink.^68^ Therefore, our assumption that calcification is the second-most important driver of ecosystem proton fluxes in this environment appears to be sound. However, in other environments where anaerobic processes play a greater role in the cycling of organic matter, anaerobic contributions to proton fluxes (including under nonsteady state conditions) need to be constrained.

### Assessment of Ecosystem Calcification and Metabolism

At the CO_2_ vent meadow, we observed net calcification during the day and at night (Figure 6d). This is physically possible as coralline algae are capable of nighttime calcification.^69,70^ However, at the control meadow we observed a more typical pattern of daytime calcification and nighttime dissolution, consistent with a tighter coupling with light.^71^ The net ecosystem calcification rates that we observed at the CO_2_ vent meadow and the control meadow were near or within the range of rates in *P. oceanica* and *Thalassia testudinum* meadows at the same time of year and at annual rates (Figure 8a).^53,72–76^ The mean rate at the CO_2_ vent meadow was one-fifth of that of a coral reef, and similar to that of a partially degraded coral reef site colonized by corallimorphs (Figure 8b).^21^

The *P. oceanica* meadows were mostly heterotrophic during these measurements. Given the seasonal decline in *P. oceanica* productivity in the autumn, this result was not surprising. The new cycle of annual growth in *P. oceanica* begins in September, but the new shoots grow into a senescent canopy.^77^ The average age of leaves in the meadow is greatest at this time of year; their carbohydrate production has been routed away from leaf maintenance, their photosynthetic maximum is low,^78^ and little light penetrates through epiphyte cover, minimizing leaf photosynthesis^79^ (though epiphytes can also contribute substantially to ecosystem photosynthesis).^47^ On four out of 5 days, the meadows were net heterotrophic (Figure 7). Taking the mean across all days, the net ecosystem metabolism was −13 ± 23 mmol O_2_ m^–2^ d^–1^. This is in the range of previous measurements of *P. oceanica* meadow productivity in August through October (+5 to −25 mmol O_2_ m^–2^ d^–1^).^53^ This rate of oxygen uptake is low compared to net oxygen production by *P. oceanica* meadows in peak summer (e.g., 77 mmol O_2_ m^–2^ d^–1^),^48^ supporting the suggestion that the carbohydrate reserves generated during the summer allow *P. oceanica* plants to sustain net heterotrophy during much of the rest of the year.^80^

The increases in daytime O_2_ production and nighttime O_2_ uptake with increases in water velocity (see O_2_ measurements in Figures S1 and S3) are likely due to multiple factors. O_2_ fluxes were weakly positively correlated with velocity, and PAR, but, in our limited data set, calcification rates had no relationships with these variables (Figure S7). Dissolved oxygen accumulation below the eddy covariance measurement volume explained, at most, half of the effect of velocity on O_2_ uptake. Mass transfer and changes in canopy structure at high water velocity may explain the remainder. Seagrass photosynthetic production can be limited by the inorganic carbon supply.^81,82^ It can also be limited by dissolved oxygen accumulation in leaf tissues, which causes photorespiration.^83^ Epiphytes can enhance these effects.^84^ At the low water velocities measured above Ischia meadows (∼2 cm s^–1^), flow in much of the canopy would have been essentially stagnant. In contrast, at the high water velocities (>10 cm s^–1^) that followed, the otherwise upright canopy is pressed toward the seafloor; leaves are bent at the rhizome, exposing their newest growth to light, and they flutter in the current, exposing more leaf surfaces to light.^85^ Similarly, for nighttime respiration, the increase in oxygen supply and decrease in diffusive boundary layer thickness may explain the increase in oxygen uptake at higher water velocities. Further work is needed to resolve this.

## Implications

This study has significant implications for the future use of pH and O_2_ measurements to investigate ecosystem calcification, and for future studies of ecosystem fluxes using boundary layer techniques at CO_2_ vents. We found that pH and O_2_ eddy covariance revealed a diurnal signal that was consistent with ecosystem calcification by coralline algae epiphytes. Further, variations in this signal were observed at time scales down to tens of minutes or less. This high temporal resolution allows boundary layer techniques to quantify continual changes in ecosystem processes with continual changes in environmental drivers, improving our understanding of the drivers of benthic ecosystem growth. We also observed patterns that invite further investigation. Differences in net calcification at the two different meadows were driven by large differences in nighttime CaCO_3_ calcification and dissolution. Increases in water velocity also appeared to drive increases in photosynthesis and respiration. Finally, we found that local sources of CO_2_, likely due to the upward diffusion of CO_2_ through sandy sediments, acidified the control meadow hundreds of meters from a known vent, resulting in the same mean pH as the CO_2_ vent meadow. Yet this upward diffusion in the vicinity of the control meadow interfered less with H^+^ and O_2_ flux measurements, potentially making it a better site to investigate the impacts of acidification on ecosystem metabolism and calcification than the CO_2_ vent meadow.

The high level of variability of calcification rates we measured, even over short time periods, illustrates the need for further development of monitoring technologies which are high frequency, high accuracy, and capable of sustained deployment. In particular, we estimate that pH sensors with an accuracy of 0.02 pH units would be required to reduce the uncertainties of future applications of O_2_ and pH techniques to measure calcification with less than 20% error. This is because organic matter respiration and production can commonly exceed calcification 5-fold (such as on coral reefs^51^). This development may allow future users to evaluate changes in benthic calcification from hour-to-hour. Those observations can resolve the environmental conditions that cause benthic calcifying ecosystems to grow and decline, improving our understanding of their future and of their management needs.

## Supplementary Material



## Data Availability

The data supporting the findings of this study are openly available at PANGAEA^86,87^ at 10.1594/PANGAEA.982880.

## Notes

Sensitivity to measurement errors (Appendix A); effect of biogeochemical processes on pH (Appendix B); complete pH and O_2_ flux time series (Figures S1, S2, and S3); sensitivity of proton production to changes in carbonate chemistry (Figure S4); O_2_ flux reproducibility (Figure S5); flux cospectra (Figure S6); and net photosynthesis and calcification as functions of PAR and water velocity (Figure S7) (PDF)
