# Supplementary material for: Benthic Ecosystem Calcification Measured with Coupled pH and O2 Aquatic Eddy Covariance
Source: ACS ES T Water. 2026 Apr 21;6(5):2719–30. doi: 10.1021/acsestwater.5c00481 (PMC13162262; doi:10.1021/acsestwater.5c00481)
Supplement: Supplementary file 1 [file ew5c00481_si_001.pdf]

Supporting Information

Benthic ecosystem calcification measured with coupled pH and O_2 aquatic eddy covariance

Dirk Koopmans[1*], Allison Schaap[1], Volker Meyer[2], Paul Färber[2], Lauren Queiss[2], Luis Montilla[3], Socratis Loucaides[1], Soeren Ahmerkamp[4], Ulisse Cardini[5]

[1]National Oceanography Centre, Southampton, SO14 3ZH, UK

[2]Max Planck Institute for Marine Microbiology, Bremen, 28359, Germany

[3]Department of Integrative Marine Biology (EMI), Stazione Zoologica Anton Dohrn, Naples, 80122, Italy

[4]Leibniz Institute for Baltic Sea Research Warnemünde, Rostock, 18119, Germany

[5]Department of Integrative Marine Biology (EMI), Stazione Zoologica Anton Dohrn – National Institute of Marine Biology, Ecology and Biotechnology, Genoa Marine Centre, Genoa, 16126, Italy

*** Correspondence:**
dirk.koopmans@noc.ac.uk

This document consists of

Appendix A: Sensitivity to Measurement Errors

Appendix B: Effect of Biogeochemical Processes on pH

Figures S1, S2, S3: Complete pH and O_2 flux time series

Figure S4: Sensitivity of proton production to changes in carbonate chemistry

Figure S5: O_2 flux reproducibility

Figure S6: Flux cospectra

Figure S7: Net photosynthesis and calcification as functions of PAR and water velocity

Appendix A: Sensitivity to Measurement Errors

We determined the sensitivity of our calculations of G_{net} to measurement error in pH and alkalinity, and to estimation errors in the net community photosynthetic quotient, PQ (i.e., $\Delta O_2/\Delta CO_2$). For these calculations, we used CO2SYS to quantify the ratio of $dH^+/dDIC$ and $dH^+/d(DIC\ 2Alk)$ at the pH values and alkalinities of interest. We calculated the total proton flux that would occur for a given G_{net} and P_{net} for the carbonate system of the seawater in this study (pH = 8.04, total alkalinity = 2660 µmol kg^{-1}, 25 °C). Specifically, if pH had been mis-measured 0.02 units low, the observer would overestimate the total proton flux, but would overestimate $dH^+/dDIC$ and $dH^+/d(DIC\ 2Alk)$ by slightly more (see Figure S6). Following Eq. 10, that mismeasurement would result in an underestimate of G_{net}. The underestimate grows exponentially where the ratio of G_{net} to P_{net} is low (Figure 7). Its growth is similar for errors in alkalinity and errors in the estimate of the net photosynthetic quotient.

We compared the underestimate of G_{net} caused by a poor estimate of the net photosynthetic quotient against the same error for the pH and O_2 gradient flux method of Takeshita et al., (2016). We first used CO2SYS to determine the effect of a given G_{net} and P_{net} on seawater pH. We then used the calculated change in pH, along with other inputs below, to solve for the change in alkalinity, following Barnes (1983) and Takeshita et al., (2016) as follows

$$-\Delta TA = \frac{(\Delta O_2 \cdot Q + (K_{z1} - K_{z2})TA_{z2} - K_{z1}(B_{z1} + OH_{z1}) + K_{z2}(B_{z2} + OH_{z2})}{(K_{z1} - 0.5)}$$

where ΔTA is the difference in total alkalinity ($TA_{z1} - TA_{z2}$), $\Delta O_2$is the difference in O_2 concentration, Q is the net photosynthetic quotient, K is the ratio of total DIC over carbonate alkalinity (using k_1 and $k_2$of Lueker et al., 2000), B is the borate concentration (Dickson, 1990), OH is the hydroxide concentration (Millero, 1995), $z1$ is close to the bed, and $z2$ is in the upper benthic boundary layer.

Literature cited

Barnes, D. J. Profiling Coral Reef Productivity and Calcification Using pH and Oxygen Electrodes. *J. Exp. Mar. Bio. Ecol.* **1983**, *66* (2), 149–161. https://doi.org/10.1016/0022-0981(83)90036-9

Dickson, A. G.; Wesolowski, D. J.; Palmer, D. A.; Mesmer, R. E. Dissociation Constant of Bisulfate Ion in Aqueous Sodium Chloride Solutions to 250 C. *J. Phys. Chem.* **1990**, *94* (20), 7978–7985. https://doi.org/10.1021/j100383a042

Lueker, T. J.; Dickson, A. G.; Keeling, C. D. Ocean pCO_2 Calculated from Dissolved Inorganic Carbon, Alkalinity, and Equations for K_1 and K_2: Validation Based on Laboratory Measurements of CO_2 in Gas and Seawater at Equilibrium. *Mar. Chem.* **2000**, *70* (1–3), 105–119. https://doi.org/10.1016/S0304-4203(00)00022-0

Millero, F. J. Thermodynamics of the carbon dioxide system in the oceans. *Geochim. Cosmochim. Acta* **1995** 59, (4), 661-677. https://doi.org/10.1016/0016-7037(94)00354-O

Takeshita, Y.; McGillis, W.; Briggs, E. M.; Carter, A. L.; Donham, E. M.; Martz, T. R.; Price, N. N.; Smith, J. E. Assessment of Net Community Production and Calcification of a Coral Reef Using a Boundary Layer Approach. *JGR Oceans* **2016**, *121* (8), 5655–5671. https://doi.org/10.1002/2016JC011886

Appendix B: The Effect of Biogeochemical Processes on pH

Soetaert et al., (2007) provide a framework that can be used to calculate the effect of biogeochemical processes on pH. Here, we use that framework to quantify the effect of sugar fermentation (represented per mole carbon as CH_2O) and the subsequent oxidation of its products via sulfate reduction. These calculations demonstrate that the net effect of fermentation, coupled sulfate reduction, and subsequent sulfide re-oxidation, is equivalent to that of oxic respiration. In other words, the dpH of Eqn. 4 plus that of Eqn. 5 equals that of Eqn. 6.

For these calculations, we used the buffer factor (pH change per increase of 1 µmol kg^{-1} of the concentration of the strong base) and mean acid-base charges that Soetaert et al., (2007) present. These values are primarily functions of pH, temperature, salinity, and pressure, which were similar enough to our conditions at Ischia to make the difference in the buffer factors less than 2.5%.

Equation 1: Fermentation

$$\mathrm{CH_2O} + \tfrac{1}{3}\mathrm{H_2O} \rightarrow \tfrac{1}{3}\mathbf{Acetate} + \tfrac{1}{3}\mathbf{CO_2} + \tfrac{2}{3}\mathrm{H_2}$$

$$\Delta\Sigma[X] = \quad (+0.333) \quad (+0.333)$$

$$\Delta X^* \quad = \quad (-1.00) \quad (-1.12)$$

$$\Delta\Sigma[-] = (-0.333) + (-0.373) = -0.707$$

$$\mathrm{dpH} = 1.32 \times 10^{-3} \times (-0.707) = -0.93 \times 10^{-3}$$

Equation 2: Acetate oxidation

$$\frac{1}{3}\mathbf{Acetate} + \frac{1}{3}\mathbf{H_2SO_4} \rightarrow \frac{2}{3}\mathbf{CO_2} + \frac{1}{3}\mathbf{H_2S}$$

$$\Delta\Sigma[-] = (+0.333) + (+0.667) + (-0.747) + (-0.323) = -0.070$$

$$\mathrm{dpH} = 1.32 \times 10^{-3} \times (-0.070) = -0.09 \times 10^{-3}$$

Equation 3: H_2 oxidation

$$\frac{2}{3}\mathrm{H_2} + \frac{1}{6}\mathbf{H_2SO_4} \rightarrow \frac{1}{6}\mathbf{H_2S} + \frac{2}{3}\mathrm{H_2O}$$

$$\Delta\Sigma[-] = (+0.333) + (-0.162) = +0.172$$

$$\mathrm{dpH} = 1.32 \times 10^{-3} \times (+0.172) = +0.23 \times 10^{-3}$$

Equation 4: Net of sulfate reduction coupled to fermentation

$$\mathrm{CH_2O} + \frac{1}{2}\mathbf{H_2SO_4} \rightarrow \mathbf{CO_2} + \frac{1}{2}\mathbf{H_2S} + \mathrm{H_2O}$$

$$\Delta\Sigma[-] = (+1.000) + (-1.120) + (-0.485) = -0.605$$

$$\mathrm{dpH} = 1.32 \times 10^{-3} \times (-0.605) = -0.80 \times 10^{-3}$$

Equation 5: Sulfide oxidation

$$\frac{1}{2}\mathbf{H_2S} + \mathrm{O_2} \rightarrow \frac{1}{2}\mathbf{H_2SO_4}$$

$$\Delta\Sigma[-] = (-0.485) + (+1.000) + = -0.515$$

$$\mathrm{dpH} = 1.32 \times 10^{-3} \times (-0.515) = -0.68 \times 10^{-3}$$

Equation 6: Oxic respiration (net of Eqns. 4 and 5)

$$\mathrm{CH_2O} + \mathrm{O_2} \rightarrow \mathbf{CO_2} + \mathrm{H_2O}$$

$$\Delta\Sigma[-] = -1.12$$

$$\mathrm{dpH} = 1.32 \times 10^{-3} \times (-1.12) = -1.48 \times 10^{-3}$$

Appendix B

Literature cited

Soetaert, K., A. F. Hofmann, J. J. Middelburg, F. J. R. Meysman, J. Greenwood. Reprint of "The effect of biogeochemical processes on pH" **2007** *Mar. Chem.* 106, (1-2), 380-401. https://doi.org/10.1016/j.marchem.2007.06.008

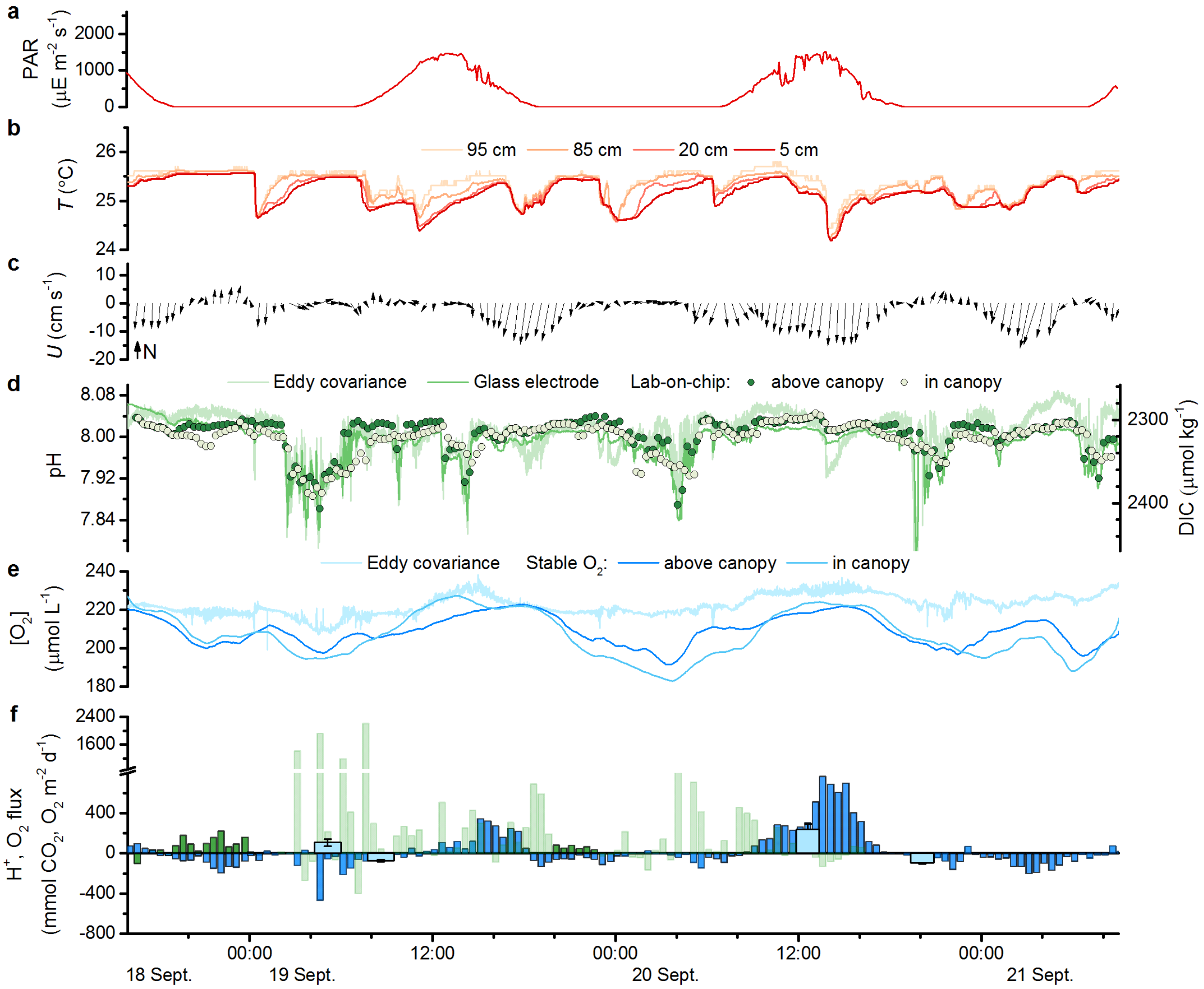

Figure S1. CO_2 vent meadow time series of (a) PAR, (b) water temperature at heights above the bed (canopy height of ~50 cm), (c) current vector with respect to north, (d) pH above and within the canopy, (e) dissolved oxygen above and within the canopy, and (f) calculated CO_2 fluxes (vent-affected present in desaturated color). The mean light and dark O_2 fluxes on 18 & 19 Sept. (combined), and on 20 Sept., are shown as wide columns in lighter blue (± s.e.).

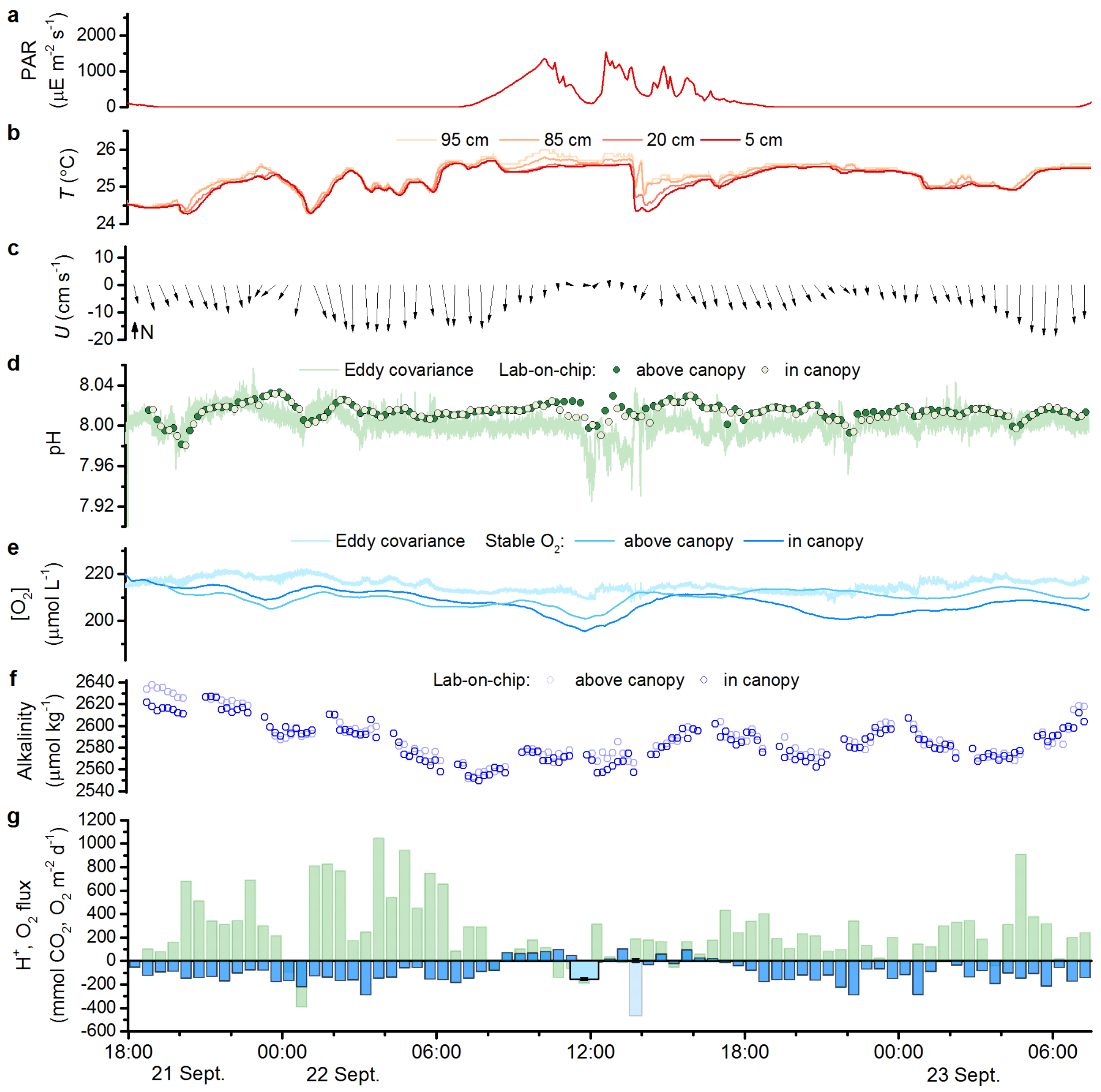

Figure S2. Second deployment at the CO_2 vent meadow. Same panels as Figure S1, except includes (f) lab-on-chip alkalinity measurements within and above the canopy. CO_2 fluxes were consistently affected by vent CO_2 during this deployment, so they were not included in calcification calculations. Transient daytime oxygen uptake (at 14:00) was caused by a change in water masses (see temperature change in panel b). It was excluded from O_2 means. The mean of the remaining light and dark O_2 fluxes on 22 September are shown. The mean of the light treatment is obscured by the y = 0 line.

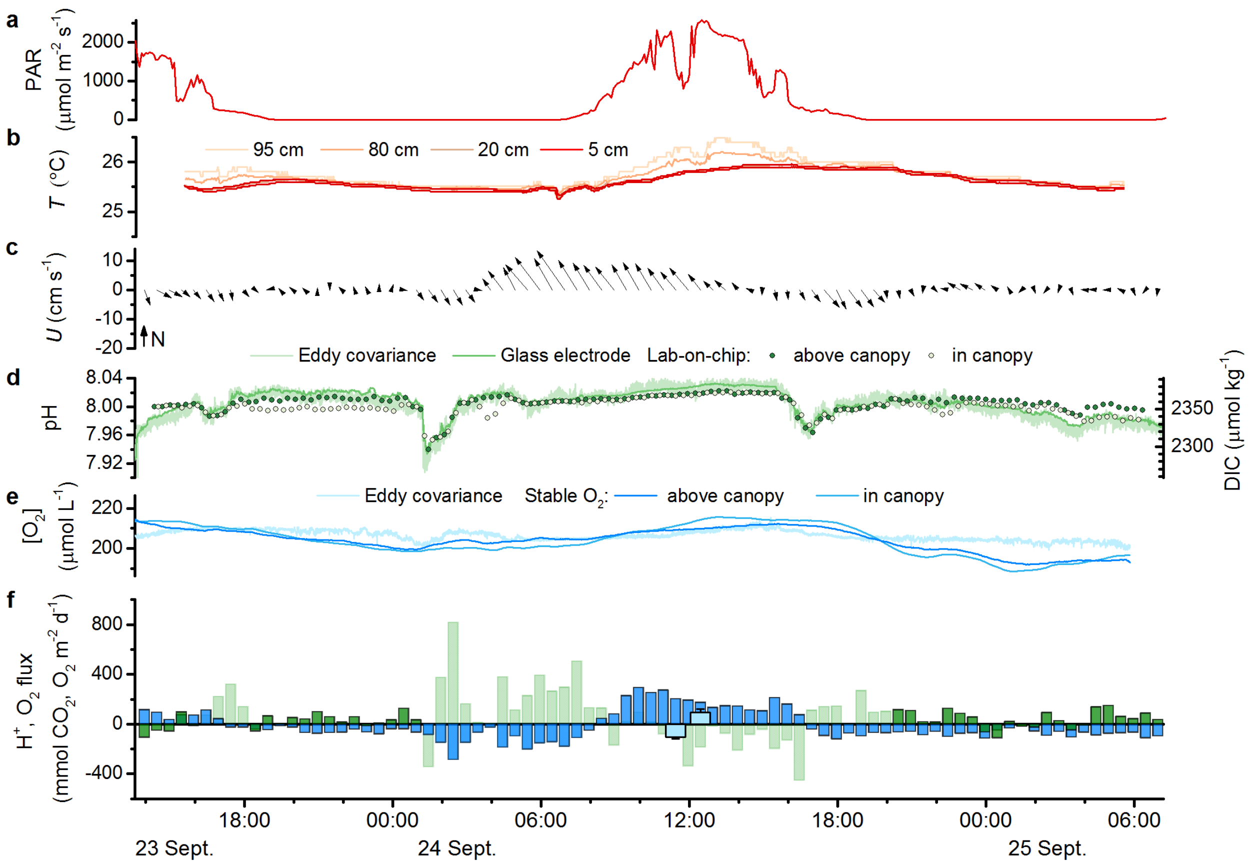

Figure S3. Control meadow time series observations. Same panels as Figure S1. The means of 24 September light and dark O_2 fluxes (± s.e.) are shown.

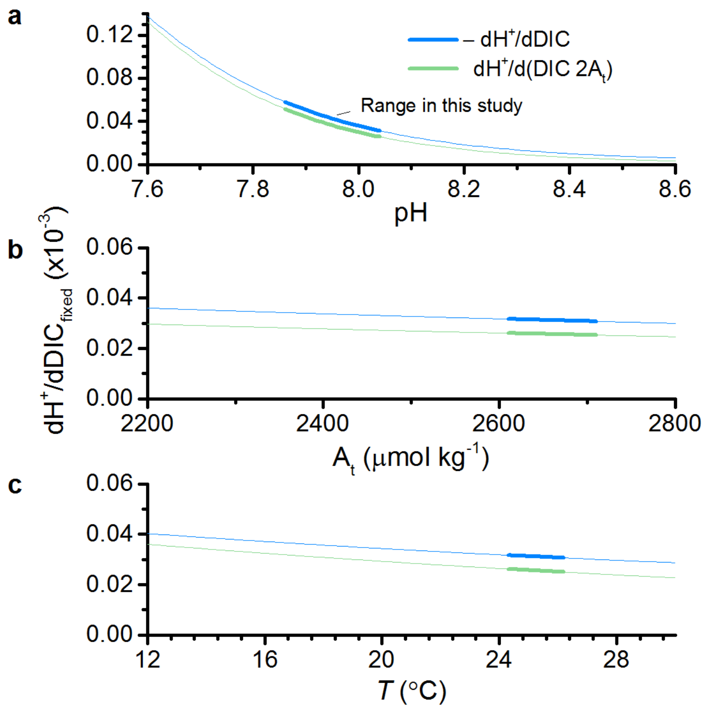

Figure S4. The sensitivity of carbonate system proton production to metabolism ($dH^+/dDIC$) and calcification ($dH^+/d(DIC\ 2A_t)$) as functions of pH, alkalinity, and temperature. $dH^+/dDIC$ quantifies μmol protons taken up per μmol of CO_2 fixed during photosynthesis (blue). $dH^+/d(DIC\ 2A_t)$ quantifies μmol protons produced per μmol of $CaCO_3$ fixed during calcification (green). Values calculated with CO2SYS (Sharp et al., 2020[38]).

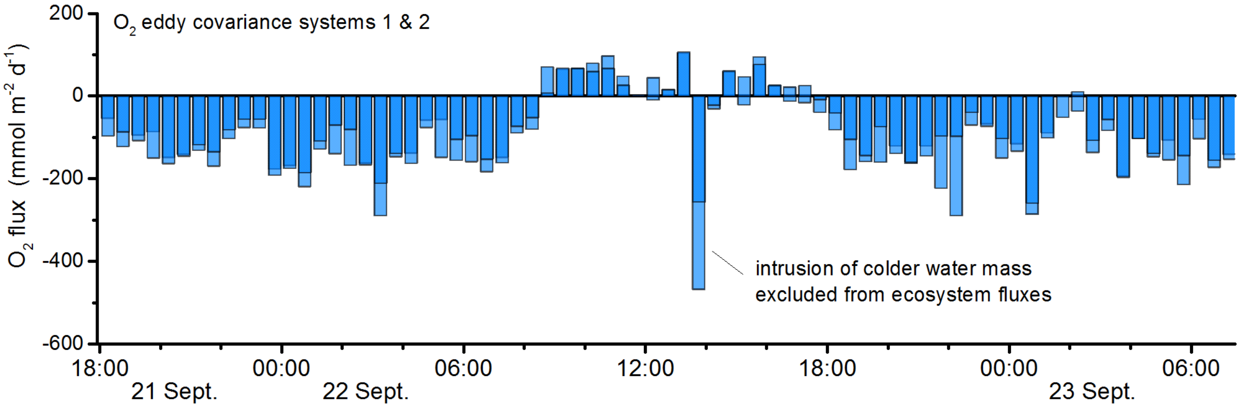

Figure S5. Example of the good agreement between dissolved oxygen fluxes measured with two independent eddy covariance systems located on separate frames at the CO_2 vent meadow. The O_2 fluxes measured by system 1 are also presented in Figure S2g.

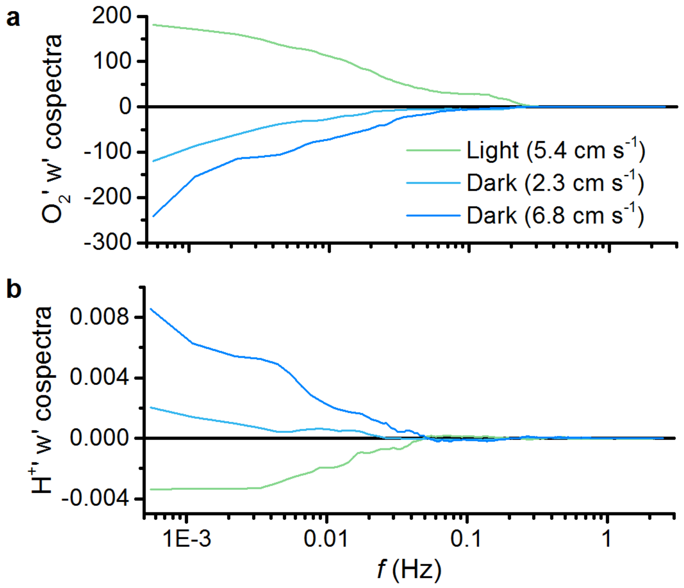

Figure S6. Cumulative cospectra of the frequencies of turbulence that contributed to measured fluxes at the control meadow. Results were similar at the CO_2 vent meadow. O_2'w' cospectra and H^+'w' cospectra were each determined over 2 h of measurement. The mean water velocities (*U*) are included in parentheses.

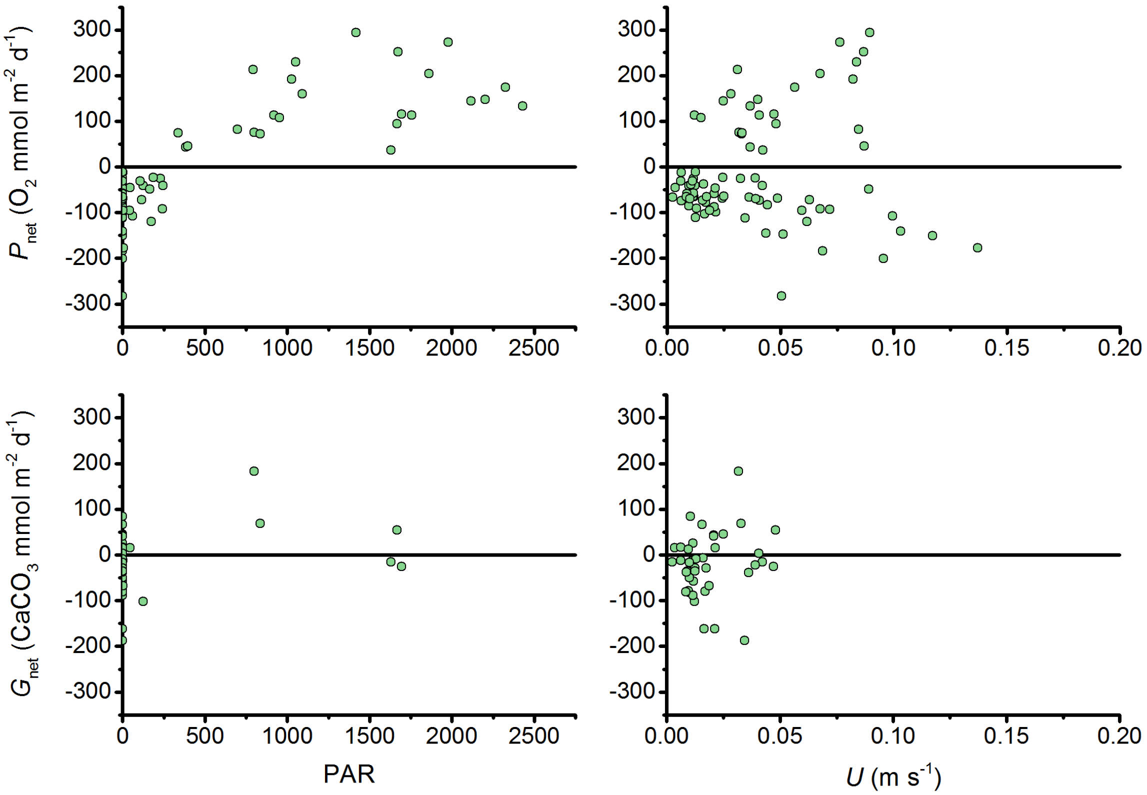

Figure S7. Ecosystem P_{net} and G_{net} expressed as functions of PAR and mean water velocity (*U*). Flux data are from the deployments during which calcification was determined (Figures S1 and S3).
